# The Beat

**Published:** 2009-03

**Authors:** Erin E. Dooley

## Arsenic Worse for Toddlers?

Arsenic contamination of drinking water is a major global health problem, with 100 million people exposed to levels above the WHO’s guideline of 10 μg/L. A study in Bangladesh has found that 18-month-old children were less able to rid their bodies of the toxic metal compared with 3-month-old breastfed infants. Reporting online 6 January 2009 in *Toxicology and Applied Pharmacology*, the researchers found a marked increase in the metabolite methylarsonic acid, which has been linked with a range of adverse health effects. The likely basis for these findings: post-weaning metabolic changes and greater intake of arsenic-tainted water and food instead of mother’s milk, which may confer some additional protection against arsenic poisoning.

**Figure:**
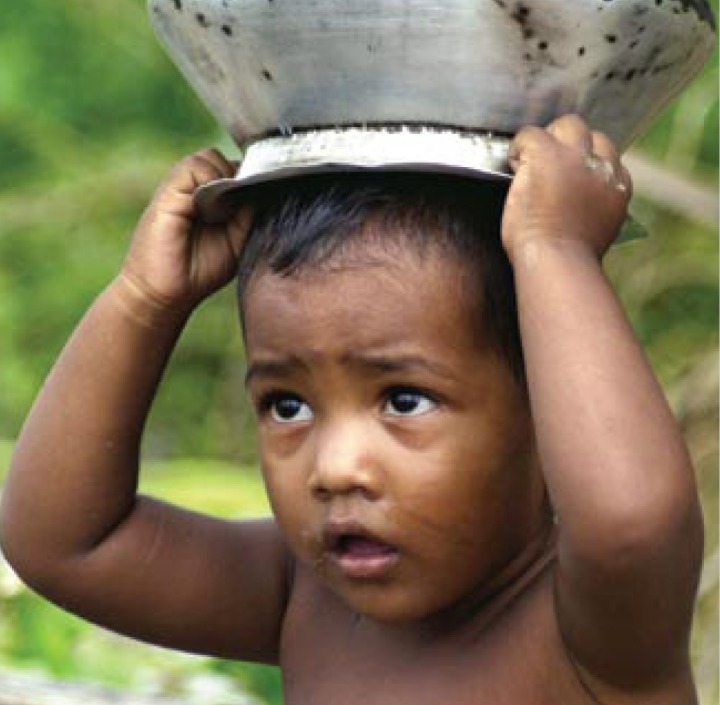
A toddler in Bangladesh

## Spicy Cleanup Option for Nanotubes

Carbon nanotubes, super-strong materials made of 1-atom-thick rolls of graphite, have outstanding electronic properties and are widely used in computers, TVs, and other products. Accidental production-related spills of nanotubes and the sheer ubiquity of these materials could someday pose a major environmental burden. Researchers reporting in the 12 November 2008 issue of *Nano Letters* now propose a “hot” solution to nanotube pollution: carbon nanotubes can be broken down by horseradish peroxidase, an enzyme long used in immunology research. Although more study is needed to refine the biodegrading process and identify potential by-products, the method has already proved successful under environmentally relevant conditions.

**Figure:**
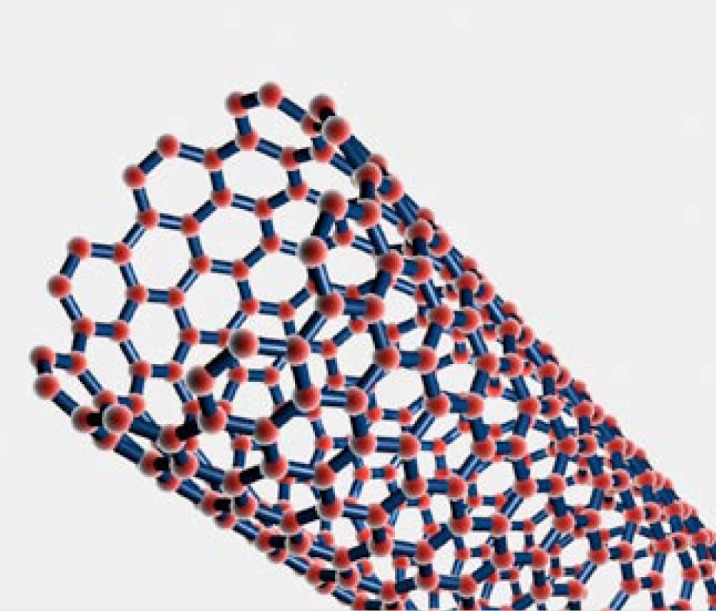
Carbon nanotubes

## NO_x_ Cap Pays Off

The 2008 annual report of the NO_x_ Budget Trading Program, a cap-and-trade partnership between federal and state governments, revealed that 2007 summertime NO_x_ emissions in 20 eastern states and the District of Columbia were down 60% from the year 2000 and down 74% from 1990. These reductions have helped curb ground-level ozone by 10% since the program’s inception in 2003. NO_x_—mixtures of nitrogen and oxygen that have been linked with climate change, acid rain, and ozone pollution—are emitted mainly by automobiles, industry, and power plants.

## Selecting Crops to Slow Warming

**Figure:**
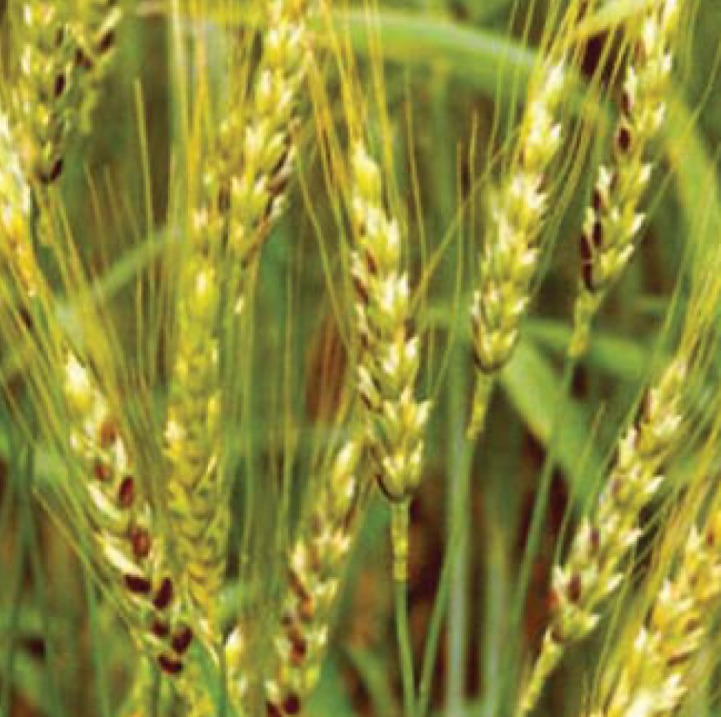
Crops such as certain wheat species are highly reflective.

Compared with natural vegetation, agricultural crops generally reflect more sunlight into space. A 27 January 2009 *Current Biology* report suggests that, by selecting crop varieties that better reflect sunlight, summer temperatures could be lowered by more than 1°C throughout much of central North America and Eurasia. The authors estimate that such a reduction would be equivalent to seasonally offsetting around 20% of the warming projected to occur in those regions by 2100. The researchers believe this low-cost intervention could temper the impact of heat waves and droughts, and that further improvements could be made by breeding more reflective plants or by genetically modifying plants to bolster their reflectivity.

## LEED Progress Report

Green construction may be paying off, according to the December 2008 *Green Building Impact Report* produced by Greener World Media. The report shows that the U.S. Green Building Council’s Leadership in Energy and Environmental Design (LEED) rating system has become a fixture of the mainstream building industry. Although the report cautions that more progress is needed if LEED is to contribute to emissions reductions in a meaningful way, initial forays are encouraging. By 2020 LEED-certified nonresidential buildings CO_2_ could result in energy savings representing about 115 million tons of avoided emissions each year. Other LEED benefits include improved indoor air quality and savings in water, land, materials, and resources.

## CA Green Chemistry Initiative

In December 2008, the California EPA debuted its Green Chemistry Initiative policy recommendations to promote the development and use of safer chemicals in the state’s industries. The initiative entails six actions: a systematic, science-based evaluation of chemicals of concern as well as their green alternatives; an online database of chemical toxicity and hazards; an online product ingredient network that discloses chemicals used in products while protecting trade secrets; a program to orient more business sectors toward pollution prevention rather than cleanup; green chemistry workforce training and education; and a system of metrics and tools for rating consumer products. It’s now up to the state to start the transition and oversee progress in the months ahead. Implementation of the first two actions will begin in 2011.

